# Smear Microscopy for Diagnosis of Pulmonary Tuberculosis in Eastern Sudan

**DOI:** 10.1155/2018/8038137

**Published:** 2018-06-14

**Authors:** Yassir A. Shuaib, Eltahir A. G. Khalil, Ulrich E. Schaible, Lothar H. Wieler, Mohammed A. M. Bakheit, Saad E. Mohamed-Noor, Mohamed A. Abdalla, Susanne Homolka, Sönke Andres, Doris Hillemann, Knut Lonnroth, Elvira Richter, Stefan Niemann, Katharina Kranzer

**Affiliations:** ^1^Research Center Borstel, Borstel, Germany; ^2^College of Veterinary Medicine, Sudan University of Science and Technology, Khartoum, Sudan; ^3^Institute of Microbiology and Epizootics, Freie Universität Berlin, Berlin, Germany; ^4^Institute of Endemic Diseases, University of Khartoum, Khartoum, Sudan; ^5^Robert Koch Institute, Berlin, Germany; ^6^Faculty of Veterinary Medicine, University of Khartoum, Khartoum, Sudan; ^7^Karolinska Institute, Stockholm, Sweden; ^8^Labor Limbach, Heidelberg, Germany; ^9^German Center for Infection Research, Borstel Site, Borstel, Germany; ^10^London School of Hygiene and Tropical Medicine, London, UK

## Abstract

**Background:**

In Sudan, tuberculosis diagnosis largely relies on clinical symptoms and smear microscopy as in many other low- and middle-income countries. The aim of this study was to investigate the positive predictive value of a positive sputum smear in patients investigated for pulmonary tuberculosis in Eastern Sudan.

**Methods:**

Two sputum samples from patients presenting with symptoms suggestive of tuberculosis were investigated using direct Ziehl-Neelsen (ZN) staining and light microscopy between June to October 2014 and January to July 2016. If one of the samples was smear positive, both samples were pooled, stored at −20°C, and sent to the National Reference Laboratory (NRL), Germany. Following decontamination, samples underwent repeat microscopy and culture. Culture negative/contaminated samples were investigated using polymerase chain reaction (PCR).

**Results:**

A total of 383 samples were investigated. Repeat microscopy categorized 123 (32.1%) as negative, among which 31 were culture positive. This increased to 80 when PCR and culture results were considered together. A total of 196 samples were culture positive, of which 171 (87.3%), 14 (7.1%), and 11 (5.6%) were* M. tuberculosis, M. intracellulare*, and mixed species. Overall, 15.6% (57/365) of the samples had no evidence of* M. tuberculosis*, resulting in a positive predictive value of 84.4%.

**Conclusions:**

There was a discordance between the results of smear microscopy performed at local laboratories in the Sudan and at the NRL, Germany; besides, a considerable number of samples had no evidence of* M. tuberculosis*. Improved quality control for smear microscopy and more specific diagnostics are crucial to avoid possible overtreatment.

## 1. Background

Worldwide, an estimated 10.4 million new tuberculosis cases occurred in 2016 with the majority of cases originating from low- and middle-income countries [1]. More than a third of these cases remained undiagnosed. Rapid and accurate diagnosis of tuberculosis is critical for timely initiation of treatment and, ultimately, control of the disease. Xpert MTB/Rif, a molecular test designed for testing clinical specimens in low-level laboratories and primary health care clinics, has changed the diagnostic landscape [2, 3]. Xpert MTB/Rif has become an integral part of tuberculosis diagnostic algorithms in many low- and middle-income countries [3–5]. However, despite the successful roll-out of Xpert MTB/Rif, smear microscopy remains the primary diagnostic tool for tuberculosis in most low-resource settings.

Sensitivity and specificity of smear microscopy for diagnosis of tuberculosis have been reported to be 30–89% and 93–100% in the context of passive tuberculosis case finding [6, 7]. Lower specificities have been reported in the context of tuberculosis prevalence surveys [8]. Some of the root causes of false positive smear microscopy results are laboratory errors such as analytic errors or sample mix-ups and the inability of smear microscopy to differentiate between* Mycobacterium tuberculosis* complex (MTBC) and nontuberculous mycobacteria (NTM). In many low- and middle-income countries, considerable efforts have been made to implement external quality assurance schemes for smear microscopy to address analytic errors [9]. Sudan, more specifically Eastern Sudan, has an external quality assurance scheme. The EQA scheme rechecks a number of randomly selected slides on a regular basis [10]. The inability of smear microscopy to differentiate between MTBC and NTM infections is further complicated by the fact that clinically and radiologically respiratory NTM infections resemble pulmonary tuberculosis. Therefore, the specificity of smear microscopy for diagnosing tuberculosis is influenced by both diagnostic quality and prevalence of NTM infections, with important implications for clinical decision-making.

In Sudan, tuberculosis diagnosis largely relies on clinical symptoms and smear microscopy as in many other low- and middle-income countries [11, 12]. More advanced diagnostics such as Xpert MTB/Rif or polymerase chain reaction (PCR) and culture are not available for routine tuberculosis diagnosis at the present time. This study aimed at investigating the positive predictive value (PPV) of Ziehl-Neelsen (ZN) smear microscopy in Eastern Sudan which is influenced by both diagnostic specificity and prevalence of the disease.

## 2. Materials and Methods

### 2.1. Study Setting

**Figure 1:**
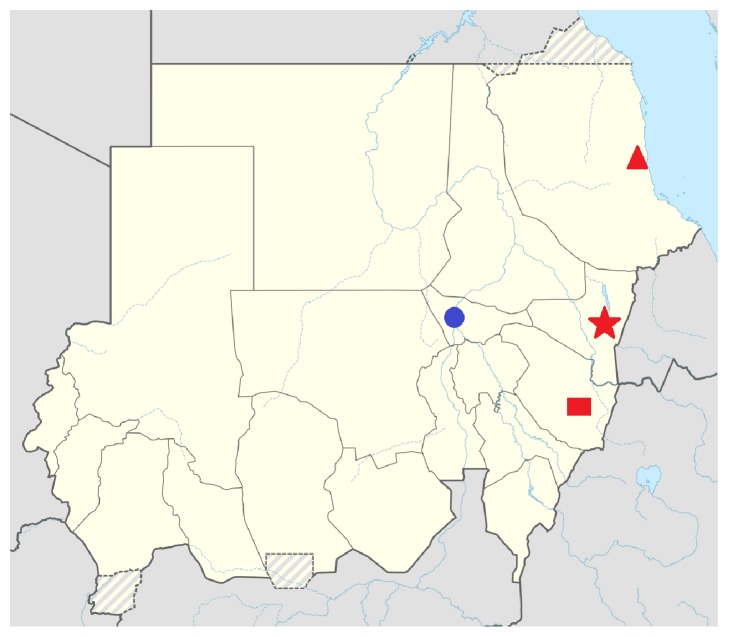
Map of the Sudan showing the three sampling sites. The capital city of the Sudan, Khartoum, is indicated by a blue circle and the three sampling sites are indicated by different red shapes. El-Gadarif is indicated by a square, Kassala is indicated by a star, and Port Sudan is indicated by a triangle.

This study was conducted in three sites in Eastern Sudan, namely, Kassala, Port Sudan, and El-Gadarif (Figure 1). Eastern Sudan shares international borders with Ethiopia, Eritrea, and Egypt and national borders with five states in the Sudan. The three selected sampling sites serve as referral clinics for tuberculosis and respiratory diseases and are very close to international borders. Approximately 600,000 people live in the catchment area of the three clinics. These people are mainly Beja, Arabs, Nubians, West Africans, and small minorities of Asians and Europeans. Between 700 and 1300 tuberculosis cases are registered every year in each of the three surveyed hospitals. Smear positivity is around 40%–50% [13–15]. Generally, HIV prevalence in Eastern Sudan was documented to be up to 0.5% while it is as high as 18.3% among tuberculosis patients [15, 16]. Overall, only 17% of all registered TB cases in the Sudan have known HIV status [1]. Eastern Sudan has witnessed an armed conflict for a long time. It ended in 2006 after signing of the peace agreement between the government and the rebel groups.

### 2.2. Study Population and Sampling

A total of 383 sputum samples of participants diagnosed with pulmonary tuberculosis on the basis of smear microscopy, clinical presentation, and chest radiography were included in this study. Patients were recruited from the outpatient department at Kassala, Port Sudan, and El-Gadarif teaching hospitals in Eastern Sudan over two recruitment periods: from June to October 2014 and from January to July 2016. Two sputum samples, a spot and an early morning, were collected from each patient. These sputum samples were subjected to direct ZN microscopy at the site of collection [6]. If at least one of the samples was smear positive, the two sputum samples were pooled, stored at −20°C, and shipped in two separate batches to the National Reference Laboratory (NRL), Borstel, Germany. The maximum period of samples storage was 6 months. All samples collected during one recruitment period were shipped together. The transit period from the Sudan to Germany was 3 days for the first batch of samples and it was longer for the second batch. All samples were shipped at room temperature.

### 2.3. Laboratory Procedures at the NRL, Germany

Samples were processed according to current guidelines by decontamination and digestion with sodium hydroxide/N-acetyl cysteine (NALC-NaOH) [17]. A portion of the resuspended sputum pellet was used for smear microscopy. Samples collected in 2014 (*n* = 101) were stained using the Kinyoun method in conjunction with an automated staining system (ZN Aerospray® TB Slide Stainer/Cytocentrifuge, Wescor, Logan, USA) and read using light microscopy (oil immersion lens, 100x). Samples collected in 2016 (*n* = 282) were stained using auramine O staining and read with a fluorescent light-emitting diode (LED) microscope (40x). Results were recorded as smear positive or smear negative. All smear positive samples were graded as scanty (+/−), 1+, 2+, and 3+, according to the World Health Organization (WHO) grading system.

The decontaminated samples were aseptically inoculated into mycobacterial growth indicator tubes (MGIT; Becton-Dickinson, Heidelberg, Germany) primed with growth supplement and antibiotics (PANTA™; Becton-Dickinson, Heidelberg, Germany). The MGIT were incubated in the BD MGIT 960 instrument for a maximum of 42 days. In addition, culture on Löwenstein-Jensen and Stonebrink slopes (own production or Enclit, Leipzig, Germany) with antibiotic supplement was performed and incubated at 37°C for a maximum of 56 days.

Positive cultures were examined microscopically for acid-fast bacilli using Kinyoun stain and inoculated on Columbia blood agar (Becton-Dickinson, Heidelberg, Germany) to rule out contamination. Identification of the grown mycobacteria was done using a commercially available line probe assay (HAIN GenoType CM and GenoType TBC; HAIN Lifescience GmbH, Nehren, Germany) and the internal transcribed spacer (ITS) sequencing. Flag-positive MGIT that demonstrated the presence of contaminants on staining and blood agar were subcultured on Löwenstein-Jensen slopes with antibiotic supplement and incubated for another 28 days at 37°C. Culture results were reported as positive for mycobacteria if there was growth on at least one of the three cultures or the subculture or negative if no growth occurred in any of the liquid and solid media, and contaminated if all three cultures and the subculture were contaminated.

Sediments which did not reveal any positive result for mycobacteria on culture in 2016 were tested using an in-house real-time PCR detecting MTBC or NTM as previously described [18]. For this, deoxyribonucleic acid (DNA) extraction was performed on the stored sputum pellets using QIAamp® DNA Minikit (QIAGEN GmbH, Hilden, Germany) as per the manufacturer's instructions.

### 2.4. Analysis

Data were entered into an Excel database. Proportions and 95% confidence intervals (CI) were calculated using the Statistical Package for the Social Sciences (SPSS) (version 20.0, SPSS Inc., Chicago, Illinois). Chi square tests were used to compare proportions.

## 3. Results

**Figure 2:**
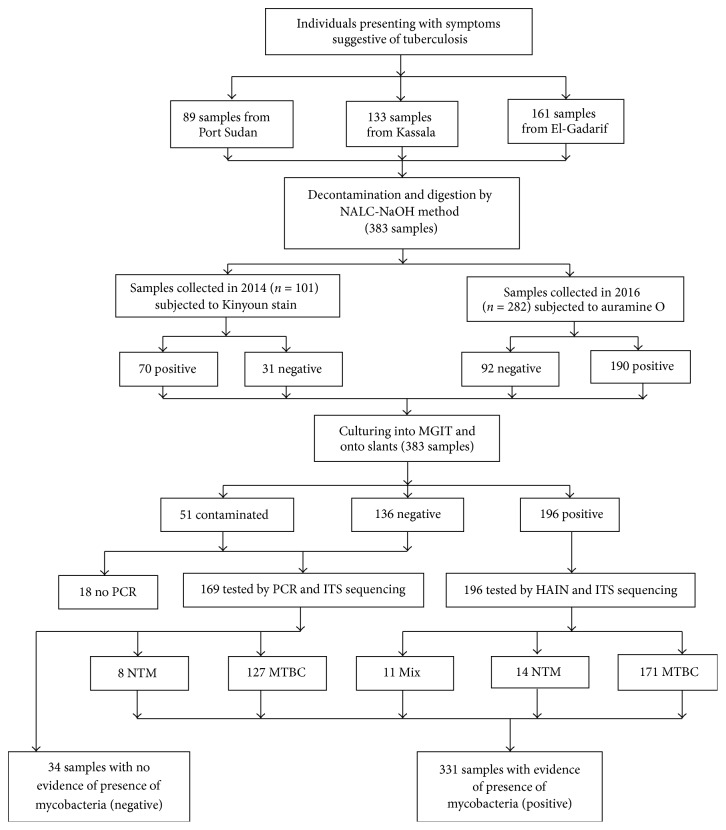
Flowchart of the results. NALC-NaOH: sodium hydroxide/N-acetyl cysteine; MGIT: mycobacteria growth indicator tube; PCR: polymerase chain reaction; HAIN: line probe assay for GenoType CM and GenoType MTBC; MTBC:* Mycobacterium tuberculosis* complex; NTM: nontuberculous mycobacteria; Mix: two different mycobacteria grown on the same culture.

A total of 383 samples were included in the analysis: 161 (42%), 133 (34.7%), and 89 (23.2%) from El-Gadarif, Kassala, and Port Sudan, respectively (Figure 2). The median age of the patients was 35 (interquartile range 25; 45). A small proportion of patients were under 15 years of age (*n* = 13, 3.4%); the majority were men (*n* = 245, 64%).

Smear scores determined in Sudan and at the NRL, Germany, were different (Figure 2). In total, one-third of the sample (*n* = 123, 32.1%) tested smear positive in Sudan were categorized as smear negative at the NRL in Germany (Table 1). Of those samples, 89 (72.4%), 22 (17.9%), and 7 (5.7%) were scored 1+, 2+, and 3+, respectively, in Sudan, while smear scores of 5 (4.0%) samples were missing.

A total of 196 (51.2%) samples revealed mycobacterial growth, while 136 (35.5%) did not show any growth and 51 (13.3%) were contaminated (Figure 2). The majority (*n* = 171, 87.3%) of mycobacterial isolates were identified as* M. tuberculosis*, 14 (7.1%) isolates were identified as* M. intracellulare* group, and 11 (5.6%) cultures were mixed, of which seven were a mixture of* M. tuberculosis* and* M. intracellulare* group, one* M. tuberculosis* and* M. fortuitum*, one* M. tuberculosis* and* M. asiaticum*, and one* M. tuberculosis* and* Corynebacterium *species, and one was a mixture of* M. intracellulare* group and an unknown* Mycobacterium* species. A total of 187 (48.8%) samples were culture negative or contaminated, of which 169 were subjected to real-time PCR and 127/169 (75.1%) tested positive for MTBC DNA and 8/169 (4.7%) for NTM DNA.

Among those samples with negative smear microscopy results (*n* = 123; 32.1%) at the NRL in Germany, 31 (25.2%) were culture positive growing MTBC. The detection rate increased to 80 (71%) samples when PCR and culture results were taken together into account (Table 1). A total of 34 culture negative samples tested by PCR did not show any evidence of mycobacterial DNA, in addition to 23 samples that had only evidence of NTM by culture or PCR. Overall, 57 (15.6%) samples had no evidence of MTBC either by culture or by PCR, compared to 308 samples for which either by culture or PCR MTBC could be proven, resulting in a positive predictive value of smear microscopy for tuberculosis of 84.4% (95% CI: 80.7–88.1) (Table 1 and Figure 2).

## 4. Discussion

This study revealed that 10% of samples categorized as smear positive in the Sudan did not have any evidence of mycobacteria using culture or PCR as reference standard in Germany. Concordance of smear scores determined by local microscopy centers in Sudan and at the NRL in Germany was suboptimal. A third of samples categorized as smear positive in Sudan were found to be negative at the NRL in Germany.

In most resource-limited settings, tuberculosis is diagnosed on the basis of smear microscopy and clinical findings. In the context of passive case finding, smear positivity is usually 10%–15%. Assuming a specificity of 98-99% and smear positivity rate of 10%–15%, positive predictive values in the range of 75%–91% would be expected (Table 2) [19]. Positive predictive values reported by African and Asian investigators vary considerably (60–98%) [20–26]. The heterogeneity of results might be explained by differences in tuberculosis prevalence and smear specificity. Unfortunately, neither prevalence nor specificity was assessed in this study, precluding any firm conclusions regarding the underlying cause of the low positive predictive value.

Generally, suboptimal smear specificity for tuberculosis is due to two reasons: (i) faulty microscopy and (ii) presence of NTMs. In this study, decreased specificity was equally attributable to reading and staining errors and the presence of NTMs. All but one of the culture and PCR negative samples (*n* = 33) were smear negative on repeat examination at the NRL. Reading and staining errors are best addressed through adequate training, regular supervision, and national external quality assurance schemes [6, 9]. Differentiation between* M. tuberculosis* and NTMs requires molecular diagnostics such as Xpert MTB/Rif or culture.

In this study, one of seven patients diagnosed with smear positive pulmonary tuberculosis at local hospitals did not have any evidence of* M. tuberculosis *bacilli in their sputum samples by culture or PCR performed in Germany. These patients were initiated on quadruple therapy with potentially toxic drugs but might not have had tuberculosis. At best, if those patients really did not have tuberculosis, they would not have benefited from treatment but would have stayed alive with untreated chronic lung disease such as bronchiectasis. At worst, those individuals would have deteriorated and/or died from their underlying disease such as lung cancer, chronic obstructive lung disease, fibrosis, or other diseases such as NTM infection. However, in low-resource settings with limited diagnostics and treatment options, there is little one can offer patients with potentially fatal lung disease with the exception of acute pneumonia and tuberculosis. This needs to be weighed against the fact that individuals receiving treatment for tuberculosis and their families will experience significant costs associated with treatment. Costs are also accrued by the health care system [27, 28].

This study has several strengths. It was a multisite study conducted in three public hospitals in Eastern Sudan, making the findings generalizable to similar settings. Cultures and molecular diagnostics were performed in a national reference laboratory in a high-resource setting. The combination of molecular diagnostics together with one liquid and two solid cultures reduced the likelihood of misclassifying a sample as negative.

The limitations of the study include lack of data on sensitivity and specificity of smear microscopy in this setting and unknown tuberculosis prevalence among individuals presenting for investigations. Furthermore, prolonged transit time might have resulted in some samples being falsely classified as culture negative. However, this was addressed by investigating culture negative samples with molecular methods. Clinical data such as HIV status, chest radiography findings, and treatment outcomes were not available. Two samples from each patient were pooled. If one of the samples was smear negative and the other one scanty smear positive, the pooled samples might be classified as smear negative at the NRL.

In summary, there was a discordance between the results of smear microscopy performed at local laboratories in the Sudan and at the NRL, Germany. Additionally, the positive predictive value of smear microscopy in Eastern Sudan was less than 90%. Half of the false positive results were due to reading and staining errors, while the other half was due to the presence of NTMs. The former needs to be addressed through quality assurance schemes, training, and supervision; the latter would require a more specific confirmatory test such as the Xpert MTB/Rif, other molecular diagnostics, or culture to avoid possible overtreatment. This is even more important in the context of active case finding when pretest probability is lower compared to passive case finding [29].

## Figures and Tables

**Table 1:** Mycobacterial culture and PCR results stratified by smear scores.

NRL smear score	Total	Culture positive for mycobacteria		Total	PCR positive for mycobacteria		Total	Culture or PCR positive for mycobacteria		Total	Culture or PCR positive for MTBC	
		Number	% (95% CI)		Number	% (95% CI)		Number	% (95% CI)		Number	% (95% CI)
Negative	123	31	25.2 (17.5–32.9)	82	49	59.8 (48.8–70.8)	113	80	71.0 (63.4–79.4)	113	70	62.0 (53.0–71.0)
Scanty	110	68	62.0 (52.9–71.1)	38	37	97.4 (95.8–99.8)	106	105	99.1 (98.3–99.9)	106	98	92.5 (87.5–97.5)
1+	77	43	55.8 (44.8–66.8)	34	34	100 (0.00-0.00)	77	77	100 (0.00-0.00)	77	74	96.0 (92.0–100)
2+	49	34	69.4 (56.4–82.4)	12	12	100 (0.00-0.00)	46	46	100 (0.00-0.00)	46	44	96.0 (93.2–99.2)
3+	24	20	83.3 (68.3–98.3)	3	3	100 (0.00-0.00)	23	23	100 (0.00-0.00)	23	22	96.0 (93.8–99.8)

Total	383	196	51.2 (46.2–56.2)	169	135	80.0 (74.0–86.0)	365	331	91.0 (87.4–95.4)	365	308	84.7 (80.7–88.1)

NRL: National Reference Laboratory.

**Table 2:** Expected positive predictive values of smear microscopy assuming a sensitivity of 55% in the context of passive case finding.

Positive predictive value	Proportion with smear positive TB	Specificity
75.3%	10%	98%
83.0%	15%	98%
87.3%	20%	98%
90.2%	25%	98%
86.0%	10%	99%
91.0%	15%	99%
93.2%	20%	99%
95.0%	25%	99%

## Data Availability

In consultation with the medical ethics committee that approved this study, the data of the study cannot be publicly available due to the privacy protection of participants. Therefore, sharing an anonymized and deidentified data set is not possible.

## Abbreviations

PPV: Positive predictive value
MTBC: * Mycobacterium tuberculosis* complex
NTM: Nontuberculous mycobacteria
PCR: Polymerase chain reaction
ZN: Ziehl-Neelsen
NRL: National Reference Laboratory
NALC-NaOH: Sodium hydroxide/N-acetyl cysteine
LED: Fluorescent light-emitting diode
WHO: World Health Organization
MGIT: Mycobacterial growth indicator tubes
DNA: Deoxyribonucleic acid
ITS: Internal transcribed spacer
CI: Confidence intervals
SPSS: Statistical Package for the Social Sciences
HIV: Human immunodeficiency virus.

## References

[1] WHO (2017). *Global Tuberculosis Report*.

[2] UNITAID, Tuberculosis Diagnostics Technology and Market Landscape, B D, Editor. 2017, WHO: Geneva, Swizerland. p. 90

[3] Sikhondze W., Dlamini T., Khumalo D. (2015). Countrywide roll-out of Xpert^®^ MTB/RIF in Swaziland: the first three years of implementation. *Public Health Action*.

[4] Gauthier M., Somoskïvi A., Berland J.-L. (2014). Stepwise implementation of a new diagnostic algorithm for multidrug-resistant tuberculosis in Haiti. *The International Journal of Tuberculosis and Lung Disease*.

[5] Van Kampen S. C., Susanto N. H., Simon S. (2015). Effects of introducing xpert MTB/RIF on diagnosis and treatment of drug-resistant tuberculosis patients in Indonesia: A pre-post intervention study. *PLoS ONE*.

[6] WHO *Approaches to improve sputum smear microscopy for tuberculosis diagnosis expert group meeting*.

[7] Cattamanchi A., Davis J. L., Pai M., Huang L., Hopewell P. C., Steingart K. R. (2010). Does bleach processing increase the accuracy of sputum smear microscopy for diagnosing pulmonary tuberculosis?. *Journal of Clinical Microbiology*.

[8] van't Hoog A. H., Meme H. K., Laserson K. F. (2012). Screening strategies for tuberculosis prevalence surveys: The value of chest radiography and symptoms. *PLoS ONE*.

[9] WHO (2003). *Quality assurance of sputum microscopy in DOTS programmes: regional guidelines for countries in the Western Pacific. Manila, Philippines*.

[10] Heldal E., FMH (2009). In-depth review of the Tuberculosis program of Sudan. *in The National Tuberculosis Programme (NTP) Sudan*.

[11] Singhal R., Myneedu V. P. (2015). Microscopy as a diagnostic tool in pulmonary tuberculosis. *International Journal of Mycobacteriology*.

[12] WHO (2016). *Sudan Tuberculosis Profile*.

[13] Abdallah T. M., Ali A. A. A. (2012). Epidemiology of tuberculosis in Eastern Sudan. *Asian Pacific Journal of Tropical Biomedicine*.

[14] Abdallah T. E. M., Toum F. E. M., Bashir O. H. (2015). Epidemiology of extra pulmonary tuberculosis in Eastern Sudan. *Asian Pacific Journal of Tropical Biomedicine*.

[15] Abdallah T. M., Siddig M. F., Ali A. A. (2011). Seroprevalence of HIV infection among tuberculosis patients in Kassala, eastern Sudan. *Journal of AIDS and HIV Research*.

[16] SNAP (2013). *Epidemiology of HIV in Sudan Staging and Analysis Sudan*.

[17] KE Kelly W., Kayes S., Matsumoto M., GLI (2014). Mycobacteriology Laboratory Manual. *Mycobacteriology Laboratory Manual*.

[18] Hillemann D., Warren R., Kubica T., Rusch-Gerdes S., Niemann S. (2006). Rapid Detection of Mycobacterium tuberculosis Beijing Genotype Strains by Real-Time PCR. *Journal of Clinical Microbiology*.

[19] Rieder H. L., Armand V. D., Kai M. K. (2007). *Priorities for Tuberculosis Bacteriology Services in Low-Income Countries*.

[20] Alfred N., Lovette L., Aliyu G. (2014). Optimising Mycobacterium tuberculosis detection in resource limited settings. *BMJ Open*.

[21] Bhalla M., Sidiq Z., Sharma P. P., Singhal R., Myneedu V. P., Sarin R. (2013). Performance of light-emitting diode fluorescence microscope for diagnosis of tuberculosis. *International Journal of Mycobacteriology*.

[22] George G., Mony P., Kenneth J. (2011). Comparison of the efficacies of loop-mediated isothermal amplification, fluorescence smear microscopy and culture for the diagnosis of tuberculosis. *PLoS ONE*.

[23] Gelaw B., Shiferaw Y., Alemayehu M., Bashaw A. A. (2017). Comparison of loop-mediated isothermal amplification assay and smear microscopy with culture for the diagnostic accuracy of tuberculosis. *BMC Infectious Diseases*.

[24] Geleta D. A., Megerssa Y. C., Gudeta A. N., Akalu G. T., Debele M. T., Tulu K. D. (2015). Xpert MTB/RIF assay for diagnosis of pulmonary tuberculosis in sputum specimens in remote health care facility Clinical microbiology and vaccines. *BMC Microbiology*.

[25] Kivihya-Ndugga L. E. A., Van Cleeff M. R. A., Githui W. A. (2003). A comprehensive comparison of Ziehi-Neelsen and fluorescence microscopy for the diagnosis of tuberculosis in a resource-poor urban setting. *The International Journal of Tuberculosis and Lung Disease*.

[26] Bonnet M., Gagnidze L., Githui W. (2011). Performance of LED-based fluorescence microscopy to diagnose tuberculosis in a peripheral health centre in Nairobi. *PLoS ONE*.

[27] Tanimura T., Jaramillo E., Weil D., Raviglione M., Lönnroth K. (2014). Financial burden for tuberculosis patients in low- and middle-income countries: a systematic review. *European Respiratory Journal*.

[28] Laurence Y. V., Griffiths U. K., Vassall A. (2015). Costs to Health Services and the Patient of Treating Tuberculosis: A Systematic Literature Review. *PharmacoEconomics*.

[29] WHO (2013). *Systematic screening for active tuberculosis: principles and recommendations*.

